# Patient information and shared decision-making in cancer care

**DOI:** 10.1038/sj.bjc.6601080

**Published:** 2003-08-15

**Authors:** A Coulter

**Affiliations:** 1 Picker Institute Europe, King's Mead House, Oxpens Road, Oxford OX1 1RX, UK

**Keywords:** decision making, patient information, oncology care

It is increasingly recognised that clinicians are not the only target audience for clinical guidelines. Patients also have a legitimate interest in learning about best practice, including evidence-based standards and treatment options. The developers of the SOR clinical practice guideline programme deserve commendation for their efforts to meet these information needs. In producing well-designed patients’ versions of the guidelines, they have set a high standard that other producers of clinical guidelines would do well to emulate.

## PATIENTS' INFORMATION NEEDS

Failure to provide sufficient information about illness and treatment is the most frequent source of patient dissatisfaction (Grol *et al*, 2000; Coulter and Cleary, 2001). It comes at the top of the list of problems identified in patient surveys and is the underlying cause of the vast majority of formal complaints and legal actions. Most cancer patients want full information about their condition and the treatment options. In a recent study of 2331 patients with different types of cancer, 98% preferred to know whether or not their illness was cancer and 87% said they wanted all possible information (Jenkins *et al*, 2001). The top three information priorities among a group of women newly diagnosed with breast cancer were (1) information about the likelihood of cure, (2) information about the spread of disease, and (3) information about treatment options (Luker *et al*, 1995). However, not all cancer patients want extensive information about their condition and treatment at all stages of their illness (Leydon *et al*, 2000). Some patients, particularly older people and men, may prefer not to delve too deeply into the details. Full information should always be offered, but health professionals must remain sensitive to patients’ varying needs.

Studies have found that patients who are well-informed about prognosis and treatment options, including benefits, harms and side effects, are more likely to adhere to treatments (Marinker *et al*, 1997). Access to evidence-based information is essential if patients are to understand the treatment options they face and if they are to participate in decisions about their care. Where there is a choice of treatments, most patients want clinicians to take account of their preferences. Patients’ preferences are not always predictable, they are sometimes discouraged from articulating them, and doctors sometimes fail to understand what patients want and why. Patients cannot express informed preferences unless they are given sufficient and appropriate information, including detailed explanations of their condition and the likely outcomes with and without treatment. They also need to be encouraged to express their concerns, beliefs, values and preferences.

## SHARED DECISION-MAKING

Although some patients prefer the doctor to make decisions on their behalf, nowadays an increasing number want to play a more active role. In this case, clinicians should offer shared decision-making in which the patient plays an equal part both in the process of decision-making and in the decision itself. The clinician must provide the patient with information about diagnosis, prognosis and treatment options, including outcome probabilities, and the patient must be prepared to clarify the level of involvement they want in the decision-making process and, if appropriate, their treatment preferences. Some cancer patients may not want to take responsibility for treatment decisions, as that implies accepting responsibility for the outcome, good or bad. The clinician must acknowledge the legitimacy of the patient's preferences and be prepared to adapt the decision-making style to accommodate what the patient wants. A number of studies have investigated the extent of desire for participation among different groups of patients. For example, in a study of 439 interactions between adult cancer patients and oncologists in an American hospital, two-thirds (69%) said they wanted to participate in treatment decisions (Blanchard *et al*, 1988). Somewhat different results were produced by a Canadian study that looked at participation preferences among 57 men with prostate cancer (Davison *et al*, 1995). The majority of these patients (58%) felt the doctor should take the primary responsibility in decision-making, 23% felt it should be an equally shared process, and 19% felt they should take the major role.

Desire for participation has been found to vary according to age, educational status, disease severity and cultural background. A study of 256 American cancer patients found that younger patients were more likely to want active participation in decisions about their care, but a substantial proportion of older patients also wanted to be involved: 87% of patients aged under 40 years expressed a desire to participate, compared with 62% of those aged 40–59 years and 51% of those aged over 60 years (Cassileth *et al*, 1980). People's preferences may vary according to the stage in the course of a disease episode and the severity of their condition. There may also be important cultural differences. For example, studies comparing responses in different countries found that British breast cancer patients were less likely to prefer an active role than Canadian ones (Richards *et al*, 1995; Beaver *et al*, 1996) and patients with colorectal cancer wanted a more passive role than patients with breast cancer (Beaver *et al*, 1999).

## DECISION AIDS

As well as determining how much information patients want, clinicians have the difficult task of finding out what role they want to play in decision-making. Given the short consultation times experienced in most busy clinics, it is often unrealistic to expect individual clinicians to provide full information about the risks and benefits of all treatment options. This information is not always readily available to doctors, let alone lay people. If patients are to be able to express their preferences, they require help in the form of user-friendly information packages or decision aids.

Research into the use of decision aids for cancer patients has shown that they can be an effective solution to these problems (O’Connor *et al*, 1999; Sowden *et al*, 2001). Decision aids help people make specific deliberative decisions about disease management and treatment options, prevention and screening. They use a variety of media to present the information in an accessible form, including leaflets, audiotapes, workbooks, decision boards, computer programs, interactive videos, web sites, structured interviews and group presentations. The content is based on reviews of clinical research and studies of patients’ information needs. They are very different from standard health information materials because they are not didactic or prescriptive – they do not tell people what to do. Instead they help patients clarify their own values and preferences and weigh up the potential benefits and harms of alternative courses of action. When participation is facilitated by using specially designed decision aids, patients’ knowledge and satisfaction with the decision process is increased (O’Connor *et al*, 2002). Clinicians who have used them with their patients report considerable benefits in terms of enhanced quality of subsequent consultations and satisfaction with the process.

Decision aids are not a substitute for good face-to-face communication between doctors and patients, but they can be a useful adjunct to the consultation. There are now more than 200 patient decision aids recorded on the Cochrane inventory (www.ohri.ca), many of which were designed for use by patients with cancer. In the process of developing and testing these, a considerable body of knowledge has been developed on how to use them to involve patients in treatment decision-making (Edwards and Elwyn, 2001). Unfortunately, there has been a reluctance to translate the lessons learnt in academic settings into the mainstream of clinical practice.

The SOR Savoir Patient booklets constitute an important addition to the range of information sources for patients. Importantly, patients were involved at all stages in the development of the booklets, greatly increasing the likelihood that the information is relevant, comprehensible and useful. What is needed now is a commitment to make this information available in oncology clinics and primary-care settings, in both paper and electronic formats so that patients and clinicians can make use of them. The booklets could be used in conjunction with a shared decision-making programme to help patients participate in decisions about which is the most appropriate treatment for them. Evidence-based clinical guidelines provide guidance, not prescription; they do not eliminate the need for judgement. Each patient is an individual and their values and preferences must be considered when deciding how to interpret the guidelines in particular cases. Patients need encouragement to express their views and clinicians need training in the communication skills and techniques required to facilitate shared decision-making. The task is not easy, but the rewards for both patient and clinician can be considerable.
